# Clinical and microbiological profiles in post-chemotherapy neutropenic fever in hematological malignancy: exploration of clinical phenotype patterns by two-step cluster analysis

**DOI:** 10.1186/s12879-023-08218-8

**Published:** 2023-04-13

**Authors:** Choi Wan Chan, Alex Molassiotis, Harold K. K. Lee

**Affiliations:** 1 School of Nursing, The Hong Kong Polytechnic University, Hong Kong, Hong Kong, Special Administrative Region of China; 2 Department of Medicine & Geriatrics, Princess Margaret Hospital, Hong Kong, Hong Kong, Special Administrative Region of China

**Keywords:** Post-chemotherapy neutropenic fever, Febrile neutropenia, Hematological malignancy, Clinical and microbiological profiles, Phenotype pattern, Two-step cluster analysis

## Abstract

**Background:**

Epidemiology of infectious diseases causing febrile illness varies geographically with human attributes. Periodic institutional surveillance of clinical and microbiological profiles in adding data to updating trends, modulating pharmatherapeutics, signifying possible excessive treatments and risk of drug resistance in post-chemotherapy neutropenic fever (NF) in hematological malignancy (HM) is limited. We aimed to review institutional clinical and microbiological data and explore clinical phenotype pattern groups among data.

**Methods:**

Available data from 372 NF episodes were included. Demographics, types of malignancies, laboratory data, antimicrobial treatments and febrile-related outcome data such as predominant pathogens and microbiological diagnosed infections (MDIs) were collected. Descriptive statistics, two-step cluster analysis and non-parametric tests were employed.

**Results:**

The occurrences of microbiological diagnosed bacterial infections (MDBIs; 20.2%) and microbiological diagnosed fungal infections (MDFIs; 19.9%) were almost equal. Gram-negative pathogens (11.8%) were comparable with gram-positive pathogens (9.9%), with gram-negative being slightly predominant. Death rate was 7.5%. Two-step cluster analysis yielded four distinct clinical phenotype pattern (cluster) groups: cluster 1 ‘lymphomas without MDIs’, cluster 2 ‘acute leukemias MDBIs’, cluster 3 ‘acute leukemias MDFIs’ and cluster 4 ‘acute leukemias without MDIs’. Considerable NF events with antibiotic prophylaxis being not identified as MDI might have cases in low-risk with non-infectious reasons causing febrile reactions that might possibly not require prophylaxis.

**Conclusions:**

Regular institutional surveillance with active parameter assessments to signify risk levels in the post-chemotherapy stage, even prior to the onset of fever, might be an evidence-based strategy in the management of NF in HM.

## Background

Patients with hematological malignancy (HM) receiving chemotherapy are rendered immunocompromised. They are at a higher rate of post-chemotherapy neutropenic fever (NF; also known as febrile neutropenia) and lethality when compared with patients with solid tumors [1–3]. Infection and mortality attributed to the post-chemotherapy NF in this population are of concern and present a challenge. Mortality rates from NF in HM vary from 3 to 39% [4, 5], with gram-negative bacterial infections associated with a high mortality rate [4]. The occurrence of gram-positive bacterial infections in the past was increased then with a decline, and gram-negative infections currently remain the predominant and most consistent cause of bacterial infections in studies [4, 6]. In addition to the epidemiology and etiology of infectious diseases causing febrile illness, which vary geographically and regionally with human attributes, a shift in the microbiological pathogenic spectrum, a change in empiric antibiotic stewardship, and widespread use of antimicrobial pharmaceuticals against causative pathogens have been reported [7, 8]. However, regular updates of institutional data per se are less frequent in HM patients – a population with immediate lethality and high rates of post-chemotherapy NF.

The pathogenic etiology is often unknown at the time of initiating empiric antimicrobial treatment. Antimicrobial prophylaxis for the high-risk hematological oncology population with prolonged days of neutropenia is recommended by international guidelines [1, 9]. However, there is also a considerable body of literature showing that the emergence of antibacterial resistance and the spread of multi-drug resistant pathogens are associated with the use antibiotic prophylaxis [10–13]. Investigators point out that the potential benefit of prophylaxis in lowering the rate of infection has been demonstrated in regions with a low to moderate antibacterial resistance rate, and that it might not be applicable to regions with a high prevalence of resistant pathogens [14–16]. There is no guarantee of the efficacy of prophylaxis related to post-chemotherapy NF, and patients can experience NF even with antimicrobial prophylaxis. In addition, post-chemotherapy NF patients with prophylaxis can experience NF due to other non-infectious causes of fever, such as drug treatment reactions or neoplastic fever [17], hence increasing the risk of drug-resistant pathogens with inappropriate use of prophylaxis. Previous research suggested that regular reviews and assessments of clinical and microbiological profiles and antibiotic policy were an important means of combatting antibiotic resistance [7, 18]. Our present study aimed to review the clinical and microbiological data of NF, and use two-step cluster analysis to explore clinical phenotype pattern (cluster) groups based on the data collected, with the comparisons of differences in clinical parameters between phenotype cluster groups.

Two-step cluster analysis, which is useful to examine the natural patterns in a set of data when the sample population is nonhomogeneous [19, 20], has been employed to identify novel cluster groups to provide complementary data for health-related issues [19, 21]. We used this analytical approach to explore NF clinical phenotype patterns by considering the important interactions that likely occur among attributes adherent to the set of data being collected, and by reducing the multidimensionality of data while discovering more relevant homogenous groups within the heterogeneous set of data in hematological malignancies.

## Methods

### Study design, population and definition an NF episode

The Research Ethics Review Committee of the hospital and the Human Subjects Ethics Review Board from the university approved the study. The study was part of a larger university and institutional review board-approved observational study investigating clinical profiles and patient-reported symptoms, and their relationships with inflammatory biomarkers and clinical prognostic data in post-chemotherapy NF patients with HMs. Patient-reported symptoms and their relationships with biomarkers and prognostic data in NF patients have been reported elsewhere [22]. This study examining clinical profiles and clustering phenotype patterns included adult patients with HMs, admitted between January 2014 and May 2019 and requiring clinical care for post-chemotherapy NF in the hematological units of a regional acute hospital. There were 372 febrile episodes between January 2014 and May 2019 – 268 and 104 febrile episodes were identified retrospectively and prospectively from January 2014 to December 2016 and June 2017 to May 2019, respectively. During the prospective period of the study, we screened 164 fever episodes. There were 155 post-chemotherapy NF episodes. Of the 155 NF episodes, 104 were in 64 patients who were enrolled in the study; therefore, these episodes were included in this study analysis. Patients who refused to join the study accounted for 51 NF episodes (32.9%). Their reasons for refusal included feeling sick, too tired or too ill, and not wanting to join the study. Informed consents were obtained from patients for the prospective collection of data. We used the same study proforma to collect and retrieve data from the patient medical records for both groups. The inclusion criteria were patients ≥ 18 years diagnosed with HM who presented with an episode of NF. NF was defined as a temperature of ≥ 38.3 °C, or of ≥ 38 °C for two episodes more than one hour apart; and an absolute neutrophil count (ANC) of < 0.5 × 109 cells/L, or of < 1 × 109 cells/L and expected to decrease below 0.5 × 109 cells/L within 48 h [23]. We defined an NF episode as the duration from the onset of NF to the point of NF subsiding (i.e. < 37.5 °C), provided that the temperature to which it subsided (i.e. < 37.5 °C) was persistent for 48 h. Subsequent episodes of fever in the same neutropenic patient were included and counted as separate, independent NF events.

### Measures of clinical parameters and data collection

In addition to demographics, types of HMs and use of empirical antibiotic treatments, we reviewed and collected laboratory data from medical records at fever presentation, including blood cell counts, biochemistry findings and inflammatory biomarkers of C-reactive protein (CRP) and procalcitonin (PCT). The normal values for PCT and CRP are taken as < 0.5 ng/mL and ≤ 5 mg/L (i.e. ≤ 5 μg/mL) respectively [24]. We also collected data for any systemic antimicrobial prophylaxis, which referred to any use of systemic antibiotic or systemic antifungal therapy within seven days before the onset of NF. Febrile-related clinical outcome parameters included data collection of microbiological diagnosed infections (MDIs), that is, microbiological diagnosed bacterial infections (MDBIs) and microbiological diagnosed fungal infections (MDFIs); presence of predominant pathogens; modifications of antibiotics during NF, if any; adverse medical complications in the first five days of and during the NF episode; total fever duration in hours; and deaths. MDIs were defined as infectious pathogen(s) detected in the laboratory specimen cultures; MDBIs and MDFIs were defined as infectious bacterial and fungal pathogen(s) respectively, detected in laboratory cultures. Laboratory tests of galactomannan assay and Beta-D-Glucan (as serum biomarkers) have the value for the diagnosis of invasive fungal infections [25, 26]. Addition to the presence of fungal pathogens detected in the laboratory cultures, we included galactomannan assay with positive result and Beta-D-Glucan level > 60 pg/ml – possible invasive fungal infection [26] in this present study to define MDFIs. Adverse medical complications, as in the previous study [27], included hypotension (systolic arterial pressure < 90 mmHg); arrhythmia; ICU admission due to septic shock; respiratory insufficiency, defined as oximetry saturation < 95% requiring oxygen therapy; documented altered mental status and acute kidney injury; and infiltrates on a chest radiograph.

### Data analysis

Descriptive statistics were used to describe the sample demographics, clinical laboratory and microbiological data, and NF clinical outcome parameters in terms of proportions, frequency, median (Med) and inter-quartile range (IQR) as appropriate. A two-step cluster analysis was used to identify clinical profile pattern (or cluster) groups within the sample data set. Given that HM is a heterogeneous population which might with changes in clinical picture and prognosis of NF events and that post-chemotherapy immunocompromised hematological patients are at risk of bacterial and fungal infections. Data on the types of HMs, MDBIs and MDFIs were input, serving as criteria for profiling the resulting clusters. The choice of a similarity measure and the determination of the number of clusters were based on the log-likelihood distance and Akaike’s information criterion (AIC) respectively. The Silhouette measure of cohesion and separation of > 0.5 as the reference value were used to indicate good quality clustering [28]. The identified model from cluster analysis was examined if it was interpretable. Differences among cluster groups were delineated descriptively, and the crosstabs chi-square, Mann–Whitney U and Kruskal–Wallis tests were used to compare data between clusters. A *p* < 0.05 level of significance was used. We did multiple comparisons among cluster groups using the Bonferroni adjustment method. Each NF episode of the total 372 episodes was taken as an individual event in the analysis of data.

## Results

### Sample demographic, clinical and microbiological profiles

This study included 372 NF episodes from 199 adult patients with HMs. Table 1 shows the summaries of sample demographic, clinical and microbiological profiles. The median age of the sample was 58 (inter-quartile range (IQR) = 17, with age ranging between 20–83 years), with 56.3% being male. Leukemias were the most common underlying malignancies, accounting for 75.8% (*n* = 282) NF episodes.

**Table 1:** Sample demographic and clinical characteristics (*n* = 372 NF episodes)

**Variables**	**Frequency (%)**
Gender	
Male	112 out of 199 (56.3)
Female	87 out of 199 (43.7)
Hematological disorders	
Myelodysplastic syndromes (MDS)	14 (3.8)
Myeloproliferative disorders (MPD)	4 (1.1)
Acute myeloid leukemia (AML)	215 (57.8)
Acute lymphocytic leukemia (ALL)	52 (14.0)
Chronic myeloid leukemia (CML)	6 (1.6)
Chronic lymphocytic leukemia (CLL)	9 (2.4)
Myeloma	12 (3.2)
Non-Hodgkin lymphoma (NHL)	57 (15.3)
Hodgkin lymphoma (HL)	3 (0.8)
Microbiological diagnosed bacterial infections (MDBIs)	75 (20.2)
Microbiological diagnosed fungal infections (MDFIs)	74 (19.9)
Pathogens	
Gram-negative bacterial pathogens	44 (11.8)
Gram-positive bacterial pathogens	37 (9.9)
Escherichia coli (E.coli)	26 (7.0)
Methicillin-resistant Staphylococcus aureus (MRSA)	19 (5.1)
Candida albicans	6 (1.6)
Enterococcus faecium	6 (1.6)
Klebsiella pneumonia	4 (1.1)
Pseudomonas aeruginosa	4 (1.1)
Enterococcus faecalis	3 (0.8)
Staphylococcus coagulase-negative	2 (0.5)
Enterobacter cloacae/enterobacter cloacae complex	2 (0.5)
Stenotrophomonas maltophilia	2 (0.5)
Candida tropicallis	2 (0.5)
Mould, yeast	2 (0.5)
Cryptococci	2 (0.5)
Aspergiollosis	1 (0.3)
Candidiasis	1 (0.3)
Candida glabrata (subtype Torulopsis glabrata)	1 (0.3)
Candida krusel	1 (0.3)
Fusariosis	1 (0.3)
Staphylococcus aureus	1 (0.3)
Streptococcus oralis	1 (0.3)
Streptococcus mitis	1 (0.3)
Clostridium species	1 (0.3)
Neisseri flarescens	1 (0.3)
Fusobacterium nucleation	1 (0.3)
Sphingobacterium species	1 (0.3)
Bacillus cereus	1 (0.3)
Coliform bacteria	1 (0.3)
Serratia marcescens	1 (0.3)
Vibrio vulnificus	1 (0.3)
Granulicate adiacens	1 (0.3)
Prophylaxis before the onset of NF	
Antibiotic	282 (75.8)
Anti-fungal	242 (65.1)
Growth factor	143 (38.4)
First-line empirical antibiotic at the onset of NF	
cefoperazone/sulbactam	185 (49.7)
piperacillin/tazobactam	111 (29.8)
With antibiotic modification during FN	245 (65.9)
Serious complications in the first 5 days of NF, during NF	
Documented chest x-ray consolidation/infiltration	68 (18.3), 80 (21.5)
Hypotension	53 (14.2), 70 (18.8)
Impaired respiratory function	52 (14), 77 (20.7)
Arrhythmia	14 (3.8), 19 (5.1)
Documented confusion/altered mental state	14 (3.8), 29 (7.8)
Severe bleeding requiring transfusion	12 (3.2), 18 (4.8)
Required admission to intensive care unit	9 (2.4), 12 (3.2)
Heart failure	5 (1.3), 7 (1.9)
Disseminated intravascular coagulation	4 (1.1), 4 (1.1)
Renal failure	3 (0.8), 8 (2.2)
Deaths	15 out of 199 patients (7.5)
AML	14
CLL	1
MDFIs	6
MDBIs	5
Gram-negative bacterial pathogens isolated	2
Gram-positive bacterial pathogens isolated	2
Gram-negative and -positive bacterial pathogens isolated	1
**Variable (range)**	**Median (IQR)**
Age (20–83 years)	58 (17)
NF duration (3–993 h)	109.5 (161.3)
ANC at the onset of NF (0–0.9 × 10^9^ cells/L)	0.0 (0.2)
Hemoglobin level at the onset of NF (1.7–13.4 g/dL) (*n* = 297)	7.9 (2)
Platelet count (1–288 × 10^9^ cells/L) (*n* = 291)	17 (29)
Albumin level (12–96 g/L) (*n* = 274)	35 (7)
Creatinine level (27–4725 µmol/L) (*n* = 281)	65 (28)
Bilirubin level (4–447 µmol/L) (*n* = 272)	15 (9)
CRP (0.9–295 mg/L) (*n* = 167)	59 (67)
CRP > 5 mg/L^a^(6–295 mg/L) (*n* = 166)	59.5 (67.8)
PCT (0.0499–318 ng/mL) (*n* = 241)	0.19 (0.32)
PCT ≥ 0.5 ng/mL^a^(0.54–318 ng/mL) (*n* = 52)	1.20 (3.85)

*NF* Neutropenic fever, *n* sample size, *IQR* Inter-quartile range, *ANC* Absolute neutrophil count, *CRP* C-reactive protein, *PCT* Procalcitonin;

^a^ ≤ 5 mg/L = CRP normal value and < 0.5 ng/mL = PCT normal value

Of the total 149 episodes (40.1%) of MDIs, there were 75 episodes (20.2%) of MDBIs and 74 episodes (19.9%) of MDFIs. Of the 74 episodes of MDFIs, they were identified by 13 NF episodes with the presence of fungal pathogen(s) – these including Candida albicans, Aspergillosis, Candidiasis, Candida tropicallis, Candida glabrata, Candida krusel, mould and yeast, Cryptococci, and Fusariosis as listed in Table 1, 69 NF episodes with Beta-D-Glucan level > 60 pg/ml and 8 NF with galactomannan assay positive result. Gram-negative pathogens were predominantly isolated (*n* = 44 episodes, 11.8%) when compared with gram-positive pathogens (*n* = 37 episodes, 9.9%). The most common pathogens isolated were Escherichia coli (E.coli) (*n* = 26 episodes, 7%), followed in decreasing order of frequency by methicillin-resistant staphylococcus aureus (MRSA) (*n* = 19 episodes, 5.1%), and other isolated pathogens as shown in Table 1.

In this sample, 15 patients died (*n* = 199, 7.5%), 14 with a diagnosis of AML and one with CLL. Six cases were MDFIs. Five were MDBIs – three with gram-negative and three with gram-positive pathogens being isolated, one of which had both gram-negative and gram-positive isolated pathogens. Documented abnormal chest x-ray (infiltration/consolidation), hypotension and impaired respiratory function were the major adverse medical complications of NF. At presentation of NF, median CRP and PCT were 59.5 mg/L and 1.2 ng/mL. Median duration of the NF was 109.5 h (or about 4.6 days).

In terms of antimicrobial prophylaxis prior to the onset of NF, there were 75.8% (*n* = 282 episodes) and 65.1% (*n* = 242 episodes) were antibiotic and anti-fungal prophylaxis respectively. Cefoperazone/sulbactam (*n* = 186, 49.7%) and piperacillin/tazobactam (*n* = 111, 29.8%) were commonly used as first-line empirical antibiotics. Over two-thirds (65.9%, *n* = 245) of the total NF episodes (*n* = 372) required antibiotic modifications after the first-line empirical antibiotic had been administered.

Further analysis of the data (Table 2) revealed no significant differences between the use of cefoperazone/sulbactam and piperacillin/tazobactam as first-line empirical antibiotics by MDBIs (*p* = 0.475) and antibiotic modification (*p* = 0.553) after administration of the respective first-line empirical antibiotic treatment (i.e. cefoperazone/sulbactam or piperacillin/tazobactam). Results of analyses show no significant differences with or without antimicrobial (antibiotic and antifungal) prophylactic treatments by MDIs (that is, MDBIs and MDFIs). Only those prescribed anti-fungal prophylaxis experienced significantly fewer complications of impaired respiratory function (*p* = 0.047) when compared with those who did not have the anti-fungal prophylaxis.

**Table 2:** First-line empirical antibiotic/antimicrobial prophylaxis by microbiological diagnosed infections, antibiotic modification required and adverse medical complications during neutropenic fever

**Clinical parameters**	**Treatments**		***P* value (Chi-square)**
	**Cefoperazone / sulbactam used in first-line empirical antibiotic (*n* = 185)** **% (*f*)**	**Piperacillin / tazobactam used in first-line empirical antibiotic (*n* = 111)** **% (*f*)**	
**MDBIs**	18.4 (34)	22.5 (25)	0.475
**Antibiotic modification required**	65.4 (121)	61.3 (68)	0.553
	**Antibiotic prophylaxis (*n* = 282)** **% (*f*)**		
	**Yes**	**No**	
**MDBIs**	20.2 (57)	20.0 (18)	1.000
**Hypotension in first five days**	13.8 (39)	15.6 (14)	0.814
**Impaired respiratory function in first five days**	12.4 (35)	18.9 (17)	0.171
**Abnormal chest x-ray in first five days**	17.0 (48)	22.2 (20)	0.340
	**Anti-fungal prophylaxis (*n* = 242)** **% (*f*)**		
	**Yes**	**No**	
**MDFIs**	19.0 (46)	21.5 (28)	0.655
**Hypotension in first five days**	14.5 (35)	13.8 (18)	0.995
**Impaired respiratory function in first five days**	11.2 (27)	19.2 (25)	0.047*
**Abnormal chest x-ray in first five days**	15.3 (37)	23.8 (31)	0.058

*n* sample size, *%* percentage of the total, *f* frequency counts, *MDBIs* microbiological diagnosed bacterial infections, *MDFIs* Microbiological diagnosed fungal infections

^*^ Crosstabs Chi-square *p* value significant at < 0.05

### Results of cluster analysis

**Fig. 1:**
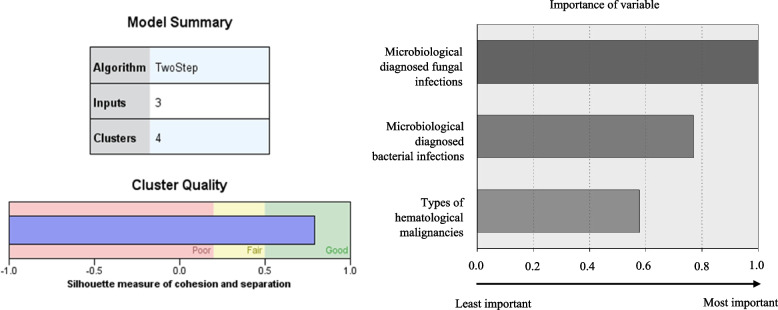
Model summary of the two-step cluster analysis, graphs of the cluster quality showing Silhouette measure of cohesion and separation of 0.8 (indicative of good quality clustering), and the importance of the three input criterion variables: microbiological diagnosed fungal infections, microbiological diagnosed bacterial infections and types of hematological malignancies in two-step cluster analysis

The best model identified by two-step cluster analysis was a four-cluster of clinical phenotype model, yielding the highest log-likelihood distance measure (ratio of distance measure = 2.5) and an AIC of 554.3 (Table 3), and producing an average Silhouette measure of cohesion and separation of 0.8, indicative of good quality clustering (Fig. 1). The identified four-cluster model was interpretable. Figure 1 shows the model summary of the two-step cluster analysis, the graphs of the quality of clusters and the importance of the three input criterion variables: types of HMs, MDBIs and MDFIs, indicating that parameters were acceptable. The sizes of the cluster groups 1, 2, 3 and 4 were formed by NF episodes of 67, 57, 74 and 174, respectively (Table 3). The ratio of largest to smallest cluster size was 3.05, which was acceptable. In the following sections, the four clinical phenotype clusters are descriptively delineated and the characteristics between clusters reported.

**Table 3:** Auto-clustering in two-step cluster analysis (yielded 4 clusters), clinical phenotype patterns and characteristics of the 4 cluster profile groups (*n* = 372)

**Auto-clustering of two-step cluster analysis**				
**Number of clusters**	**AIC**	**AIC changes **^a^	**Ratio of AIC changes **^b^	**Ratio of distance measures **^c^
1	1462.026			
2	1082.597	-379.429	1.000	1.394
3	814.276	-268.321	.707	1.030
*4	**554.304**	**-259.972**	**.685**	**2.497**
5	458.562	-95.742	.252	1.240
6	384.059	-74.503	.196	1.546
7	340.809	-43.251	.114	1.168
8	305.787	-35.022	.092	1.248
9	280.495	-25.292	.067	1.171
10	260.953	-19.542	.052	1.207
11	247.160	-13.794	.036	1.182
12	237.652	-9.508	.025	1.162
13	231.420	-6.232	.016	1.636
14	233.054	1.635	-.004	1.079
15	235.597	2.543	-.007	1.278
**Clinical phenotype patterns and characteristics of the 4 cluster profile groups (*n* = 372)**				
	**Cluster** **n (%)**			
**Clinical characteristics (n)**	**1** **67 (18)**	**2** **57 (15.3)**	**3** **74 (19.9)**	**4** **174 (46.8)**
Types of hematological malignancies				
MDS (*n* = 14)	9 (64.3)	0 (0)	5 (35.7)	0 (0)
MPD (*n* = 4)	1 (25)	1 (25)	2 (50)	0 (0)
Acute leukemias: AML & ALL (*n* = 267)	0 (0)	46 (17.2)	47 (17.6)	174 (65.2)
Chronic leukemias: CML & CLL (*n* = 15)	8 (53.3)	1 (6.7)	6 (40)	0 (0)
Myeloma (*n* = 12)	9 (75)	3 (25)	0 (0)	0 (0)
Lymphomas: NHL & HL (*n* = 60)	40 (66.7)	6 (10)	14 (23.3)	0 (0)
Microbiological diagnosed fungal infections (*n* = 74)	0 (0)	0 (0)	74 (100)	0 (0)
Microbiological diagnosed bacterial infections (*n* = 75)	0 (0)	57 (76)	18 (24)	0(0)
Deaths (*n* = 15)	0 (0)	2 (13.3)	6 (40)	7(46.7)
AML (*n* = 14)	0 (0)	2 (14.3)	5 (35.7)	7 (50)
CLL (*n* = 1)	0 (0)	0 (0)	1 (100)	0 (0)

*AIC* Akaike’s Information Criterion, *n* cluster (sample) size in NF episodes, *%* cluster size/distribution in percentages, *MDS* Myelodysplastic syndromes, *MPD* Myeloproliferative disorders, *AML* Acute myeloid leukemia, *ALL* Acute lymphocytic leukemia, *CML* Chronic myeloid leukemia, *CLL* Chronic lymphocytic leukemia, *NHL* Non-Hodgkin lymphoma, *HL* Hodgkin lymphoma

^*^ And bolded-text of the row = The best model identified is a 4-cluster model, yielding the highest log-likelihood distance measure (ratio of distance measure = 2.497), an AIC of 554.304, an average Silhouette measure of cohesion and separation of 0.8 (in Fig. 1, indicative of good quality clustering), and this 4-cluster model was interpretable

^a^ The changes are from the previous number of clusters in the table

^b^ The ratios of changes are relative to the change for the two-cluster solution;

^c^ The ratios of distance measures are based on the current number of clusters against the previous number of clusters

### Clinical phenotype patterns and characteristics of clusters (Tables 3 & 4)

**Table 4:** Comparison of differences in clinical characteristics/parameters between clusters

**Clinical characteristics (n)**	**Cluster**				***Compare 4-cluster p*** ** value (Chi-square)**	**Comparison of differences between pairs of clusters**	***Compare pairwise 2-cluster p*** ** value (Chi-square)**
	**1**	**2**	**3**	**4**			
	**Count (%)**						
Microbiological diagnosed fungal infections (MDFIs) (*n* = 74)	0(0)	0(0)	74(100)	0(0)	< 0.0001*	Clusters 1 & 2	–-
						Clusters 1 & 3	< 0.0001*
						Clusters 1 & 4	–-
						Clusters 2 & 3	< 0.0001*
						Clusters 2 & 4	–-
						Clusters 3 & 4	< 0.0001*
Microbiological diagnosed bacterial infections (MDBIs) (*n* = 75)	0(0)	57(76)	18(24)	0(0)	< 0.0001*	Clusters 1 & 2	< 0.0001*
						Clusters 1 & 3	< 0.0001*
						Clusters 1 & 4	–-
						Clusters 2 & 3	< 0.0001*
						Clusters 2 & 4	< 0.0001*
						Clusters 3 & 4	< 0.0001*
Presence of gram-positive bacteria (*n* = 37)	0(0)	25(67.6)	12(32.4)	0(0)	< 0.0001*	Clusters 1 & 2	< 0.0001*
						Clusters 1 & 3	0.002*
						Clusters 1 & 4	–-
						Clusters 2 & 3	0.001*
						Clusters 2 & 4	< 0.0001*
						Clusters 3 & 4	< 0.0001*
Presence of gram-negative bacteria (*n* = 44)	0(0)	35(79.5)	9(20.5)	0(0)	< 0.0001*	Clusters 1 & 2	< 0.0001*
						Clusters 1 & 3	0.009*
						Clusters 1 & 4	–-
						Clusters 2 & 3	< 0.0001*
						Clusters 2 & 4	< 0.0001*
						Clusters 3 & 4	< 0.0001*
Presence of Escherichia coli (E.coli) (*n* = 26)	0(0)	23(88.5)	3(11.5)	0(0)	< 0.0001*	Clusters 1 & 2	< 0.0001*
						Clusters 1 & 3	0.279
						Clusters 1 & 4	–-
						Clusters 2 & 3	< 0.0001*
						Clusters 2 & 4	< 0.0001*
						Clusters 3 & 4	0.042*
Presence of Methicillin-resistant Staphylococcus aureus (MRSA) (*n* = 19)	0(0)	12(63.2)	7(36.8)	0(0)	< 0.0001*	Clusters 1 & 2	< 0.0001*
						Clusters 1 & 3	0.028*
						Clusters 1 & 4	–-
						Clusters 2 & 3	0.106
						Clusters 2 & 4	< 0.0001*
						Clusters 3 & 4	< 0.0001*
Anti-microbial prophylaxis before the onset of NF							
Anti-bacterial (antibiotic) (*n* = 282)	48(17)	42(14.9)	57(20.2)	135(47.9)	0.769	Clusters 1 & 2	0.958
						Clusters 1 & 3	0.590
						Clusters 1 & 4	0.424
						Clusters 2 & 3	0.813
						Clusters 2 & 4	0.672
						Clusters 3 & 4	1.000
Anti-fungal (*n* = 242)	35(14.5)	40(16.5)	46(19)	121(50)	0.067	Clusters 1 & 2	0.064
						Clusters 1 & 3	0.308
						Clusters 1 & 4	0.018*
						Clusters 2 & 3	0.440
						Clusters 2 & 4	1.000
						Clusters 3 & 4	0.324
Antibiotic modification during FN (*N* = 245)	36(14.7)	43(17.6)	57(23.3)	109(44.5)	0.009*	Clusters 1 & 2	0.020*
						Clusters 1 & 3	0.006*
						Clusters 1 & 4	0.263
						Clusters 2 & 3	0.996
						Clusters 2 & 4	0.108
						Clusters 3 & 4	0.040*
Medical complications							
Hypotension in first five days (*n* = 53)	4(7.5)	16(30.2)	12(22.6)	21(39.6)	0.004*	Clusters 1 & 2	0.002*
						Clusters 1 & 3	0.099
						Clusters 1 & 4	0.248
						Clusters 2 & 3	0.154
						Clusters 2 & 4	0.008*
						Clusters 3 & 4	0.499
Hypotension during NF event (*n* = 70)	5(7.1)	17(24.3)	18(25.7)	30(42.9)	0.008*	Clusters 1 & 2	0.003*
						Clusters 1 & 3	0.013*
						Clusters 1 & 4	0.084
						Clusters 2 & 3	0.613
						Clusters 2 & 4	0.063
						Clusters 3 & 4	0.264
Respiratory failure in first five days (*n* = 52)	7(13.5)	13(25)	16(30.8)	16(30.8)	0.010*	Clusters 1 & 2	0.105
						Clusters 1 & 3	0.118
						Clusters 1 & 4	0.959
						Clusters 2 & 3	1.000
						Clusters 2 & 4	0.014*
						Clusters 3 & 4	0.014*
Respiratory failure during NF event (*n* = 77)	10(13)	16(20.8)	27(35.1)	24(31.2)	< 0.0001*	Clusters 1 & 2	0.116
						Clusters 1 & 3	0.007*
						Clusters 1 & 4	0.984
						Clusters 2 & 3	0.407
						Clusters 2 & 4	0.023*
						Clusters 3 & 4	< 0.0001*
Abnormal chest x-ray in first five days (*n* = 68)	8(11.8)	14(20.6)	20(29.4)	26(38.2)	0.039*	Clusters 1 & 2	0.110
						Clusters 1 & 3	0.042*
						Clusters 1 & 4	0.694
						Clusters 2 & 3	0.906
						Clusters 2 & 4	0.143
						Clusters 3 & 4	0.039*
Abnormal chest x-ray during NF event (*n* = 80)	10(12.5)	16(20)	25(31.3)	29(36.3)	0.007*	Clusters 1 & 2	0.116
						Clusters 1 & 3	0.017*
						Clusters 1 & 4	0.894
						Clusters 2 & 3	0.611
						Clusters 2 & 4	0.090
						Clusters 3 & 4	0.005*
**Clinical characteristics (n)**	**Cluster**				***Compare 4-cluster p*** ** value (K-W test)**	**Comparison of differences between pairs of clusters**	***Compare pairwise 2-cluster p*** ** value (MWU test)**
	1	2	3	4			
	Median (IQR)						
Age (*n* = 372)	56 (19)	57(19)	63(16)	58(17)	0.016*	Clusters 1 & 2	0.369
						Clusters 1 & 3	0.004**
						Clusters 1 & 4	0.138
						Clusters 2 & 3	0.036
						Clusters 2 & 4	0.719
						Clusters 3 & 4	0.017
NF duration in hours (*n* = 372)	79 (118)	129 (137)	192 (299.6)	88.8 (142.5)	< 0.0001*	Clusters 1 & 2	0.004**
						Clusters 1 & 3	< 0.008**
						Clusters 1 & 4	0.356
						Clusters 2 & 3	0.046
						Clusters 2 & 4	0.010
						Clusters 3 & 4	< 0.008**
Laboratory parameters at onset of NF							
Absolute neutrophil count (ANC) (*n* = 372)	0(0.2)	0(0.1)	0.1(0.5)	0(0.2)	0.028*	Clusters 1 & 2	0.053
						Clusters 1 & 3	0.332
						Clusters 1 & 4	0.499
						Clusters 2 & 3	0.004**
						Clusters 2 & 4	0.104
						Clusters 3 & 4	0.050
Hemoglobin (Hb) (*n* = 297)	8.7(3)	7.5(2.1)	8.2(2)	7.7(1.8)	0.004*	Clusters 1 & 2	0.021
						Clusters 1 & 3	0.114
						Clusters 1 & 4	0.001**
						Clusters 2 & 3	0.160
						Clusters 2 & 4	0.915
						Clusters 3 & 4	0.031
Platelet count (*n* = 291)	36(91)	13(12.5)	15(24)	17(27)	0.001*	Clusters 1 & 2	< 0.008**
						Clusters 1 & 3	0.013
						Clusters 1 & 4	0.007**
						Clusters 2 & 3	0.116
						Clusters 2 & 4	0.010
						Clusters 3 & 4	0.501
Albumin (*n* = 274)	35(8)	35(7.8)	33(8.3)	35(6)	0.054	–-	–-
Creatinine (*n* = 281)	66 (29.3)	65 (26)	66.5 (35.3)	64 (25)	0.269	–-	–-
Bilirubin (*n* = 272)	15(10)	15(12)	15(15.8)	14(9)	0.523	–-	–-
CRP (*n* = 167)	49.5 (96.8)	57 (42.5)	67 (100.5)	65 (62.3)	0.844	–-	–-
CRP > 5 mg/L^a^ (*n* = 166)	50 (96.5)	57 (42.5)	67 (100.5)	65 (62.3)	0.904	–-	–-
PCT (*n* = 241)	0.22 (0.35)	0.24 (1.05)	0.29 (0.43)	0.16 (0.19)	0.019*	Clusters 1 & 2	0.495
						Clusters 1 & 3	0.477
						Clusters 1 & 4	0.080
						Clusters 2 & 3	0.944
						Clusters 2 & 4	0.023
						Clusters 3 & 4	0.011
PCT ≥ 0.5 ng/mL^a^(*n* = 52)	1.32 (4.1)	4.55 (12.63)	0.87 (1.55)	0.97 (1.01)	0.146	–-	–-

*n* sample size, *%* percentage of the total, *K-W test* Kruskal–Wallis test, *MWU test* Mann–Whitney U test, *CRP* C-reactive protein, *PCT* Procalcitonin

^a^ ≤5 mg/L=CRP normal value and <0.5 ng/mL=PCT normal value

^*^ *P* value significant at < 0.05

^**^ *P* value significant at < 0.008 after a Bonferroni adjustment to the alpha value of 0.05

#### Cluster 1 (*n* = 67 episodes)

The cluster consisted of NF episodes, predominantly lymphoma cases, with no documented MDIs, and was labeled the ‘lymphomas without MDIs’ cluster. No deaths were reported from this cluster. Cluster 1 was the youngest profile group in terms of age (Med = 56 years old).

#### Cluster 2 (*n* = 57 episodes)

Cluster 2 consisted of NF episodes, with the majority being acute leukemia cases that were predominant with MDBIs. It was labeled the ‘acute leukemias MDBIs’ cluster. A majority of the MDBIs (76%, *n* = 57) were in this cluster. Two deaths from AML were documented, giving a death rate of 3.5% in this cluster. These deaths were MDBIs, one with gram-negative isolated pathogens and the other with gram-positive pathogens. This cluster was the smallest group of the four clusters.

#### Cluster 3 (*n* = 74 episodes)

Cluster 3 consisted of NF episodes; the majority of acute leukemia cases, all MDFIs (*n* = 74) and 24% MDBIs (*n* = 18) were documented in this cluster, which was predominantly labeled the ‘acute leukemia MDFIs’ cluster. There were six deaths, five AML and one CLL, giving a death rate of 8.1% within this cluster – a mixed group of MDBIS and MDFIs, and the highest death rate group when compared with the other three cluster groups. The six deaths were all MDFIs. Of them, three cases were also MDBIs, including one with gram-negative isolated pathogens, one with positive pathogens, and one (CLL) with both gram-negative and gram-positive pathogens being isolated. Cluster 3 was the oldest profile group in terms of age (63 years old).

#### Cluster 4 (*n* = 174 episodes)

This cluster of NF episodes was all acute leukemia cases, with no MDIs; it was labeled the ‘acute leukemias without MDIs’ cluster. The death rate within this cluster was 4%, with seven AML deaths documented. It was the largest cluster, with two to three times more NF episodes than the other clusters.

### Comparing differences in clinical characteristics/parameters by cluster (Table 4)

We compared clinical parameters across clusters. Results showed significant differences in parameters by cluster, including age, fever duration, ANC, hemoglobin level, platelet count, presence of MDFIs, MDBIs, gram-negative bacterial pathogens, gram-positive bacterial pathogens, E.coli, MRSA, modification of antibiotics required during NF event, and adverse medication complications in the first five days of and during the NF event. Clinical parameters resulted in a non-significant difference across clusters that included antimicrobial prophylaxis prior to NF onset, biochemistry data of albumin, creatinine and bilirubin levels, and inflammatory biomarkers of CRP and PCT.

Cluster 2, ‘acute leukemia MDBIs’, had the highest proportions of bacterial pathogens compared with the other three cluster groups: 79.5% (*n* = 35) gram-negative pathogens, 67.6% (*n* = 25) gram-positive pathogens, 88.5% (*n* = 23) E.coli and 63.2% (*n* = 12) MRSA respectively. Significantly longer fever duration was found in cluster 3, ‘acute leukemia MDFIs’ (8 febrile days). Cluster 4, ‘acute leukemias without MDIs’, had significantly higher proportions of NF episodes requiring antibiotic modifications after the initial first-line empirical antibiotic treatment when compared with the other three clusters (*n* = 109 episodes, *p* = 0.009). Regarding adverse medical complications in the first five days of NF, cluster 4, ‘acute leukemias without MDIs’, had significantly higher rates of hypotension (*p* = 0.004), impairment of respiratory function (*p* = 0.01) and abnormal chest x-ray (*p* = 0.039) than the other three clusters. Further details of post hoc analyses on parameters are shown in Table 4.

In terms of laboratory parameters, pairwise comparisons of cluster 1, ‘lymphomas without MDIs’, having a significant high in median hemoglobin level and platelet count, when compared with other clusters are shown (Table 4).

## Discussion

A considerable sample size of 372 NF in post-chemotherapy HM in our study adds recent knowledge to complement previous limited surveillance studies about the trends of clinical and microbiological profiles. Furthermore, using two-step cluster analysis to explore NF clinical phenotype profile pattern groups in post-chemotherapy HM was unlike previous studies, in which clinical phenotype cluster groups of clinical and microbiological characteristics were less investigated [4, 5, 29–31]. Distinct phenotype profile groups identified by cluster analysis reduced the multidimensionality among the heterogeneous HM population with post-chemotherapy NF, which might enhance our knowledge to focusing meaningful profile patterns for appropriate therapeutic management in order to improve antimicrobial clinical outcomes.

Microbiological isolates in about 40% of MDIs showed that our finding was within range when compared with those reported in the literature, which vary from 35.39% to over 56% [4, 5]. The disparity of isolates between gram-negative bacteria (11.8%) and gram-positive bacteria (9.9%) was modest in our study compared with previous research that was markedly lower in gram-positive bacteria (e.g. as low as 15%) in comparison with the predominantly high gram-negative bacteria (e.g. as high as 85%) [4, 5, 30]. Among the five deaths (out of a total 15 deaths) reported in the present study, two had gram-negative bacteria, two gram-positive, and one had both gram-negative and gram-positive bacteria being isolated. MRSA was 5.1%, which was comparable with a previous study [5] and contributed the second most common isolates in our study. Gram-positive bacterial infections in our institution would not be overlooked, even though they were less prevalent but with a smaller disparity in relation to gram-negative bacterial infections. Consistent with many surveillance studies [4, 5, 30, 32], E.coli (7%) was the most common gram-negative bacterial pathogen isolated in our surveillance. Mortality in the HM population is reported variably in the literature; the 7.5% mortality rate in our study was comparable to the 8% mortality reported in a previous study [7], but not to others with reported rates of 3% and 39% [4, 5]. The occurrences of MDBIs and MDFIs were almost equal. In addition, there were six deaths with MDFIs compared with five deaths with MDBIs in the present study, echoing the literature in which fungal infection has been a major infective cause of mortality in HM, besides the threats posed by bacterial infections, which have receded to some extent [33].

All MDIs (100%) and more than 50% of adverse medical complications and mortality were clustered with acute leukemia and lymphoma populations from cluster 2, ‘acute leukemias MDBIs’, and cluster 3, ‘acute leukemias MDFIs’, respectively. Two-step cluster analysis might reveal meaningful phenotype patterns because it clearly identified clusters 2 and 3 with one-third of acute leukemia population and one-third of lymphoma population, which might be of key concern for critical management of post-chemotherapy NF. Given that we found no significant differences with and without antibiotic prophylaxis by adverse medical complications (Table 2), and that > 77.5% (135/174) of NF in cluster 4 and > 71.6% (48/67) of NF in cluster 1 with antibiotic prophylaxis were found without microbiological diagnosed infections, there were concerning issues that we might not overlook. These issues in fact were challenges that might require future investigations, including (1) if there were any inappropriate use/overuse of prophylaxis; (2) if those cases were possible clinically diagnosed infections in situations where microbiological isolates in the laboratory cultures were not being identified; and (3) if those were low-risk cases with merely drug treatment effect causing febrile reactions that would possibly not require prophylaxis. Furthermore, although two-step cluster analysis in this study revealed meaningful phenotype patterns, further validation of the model phenotype patterns in future research using an independent sample or other alternative validating approaches might be central for clinical practice to manage HM population with high rates of post-chemotherapy NF – yet, that’s a challenge.

Consistent with guideline recommending anti-fungal prophylaxis used for high-risk patients with prolong neutropenia [9], anti-fungal prophylaxis was required in FN management in HM in our results, which showed that anti-fungal prophylaxis significantly reduced the medical complication of impaired respiratory function (Table 2, *p* = 0.047). Fungal infections other than bacterial infections, have been escalated as a major infective cause of mortality and found in a significant incidence in the HM population, as documented in the literature [33, 34].

It is noteworthy that cluster 4, ‘acute leukemias without MDIs’, accounted for 4% of mortality within its cluster (7/174 deaths). Clinically diagnosed infections in the absence of identified isolates might be plausible in cluster 4. Across clusters, cluster 4 had the highest proportion of NF events with adverse medical complications (> 39% hypotension, > 30% impaired respiratory function), with relatively high antibiotic modifications (44.5%) compared with other clusters, median Hb level and platelet count in the lower end of the range limits, and abnormal median inflammatory biomarkers values indicating of systemic severe bacterial sepsis. Rather/other than widely administering prophylaxis, frequent monitoring of patient vital sign conditions (parameters) and active checking of laboratory data, including cultures for isolates, blood cell counts and inflammatory biomarkers in the post-chemotherapy stage, even prior to the onset of NF during the course of institution surveillance, might be an alternative approach to combat antibiotic/drug resistance and for clinically diagnosed infections. We consider this approach promising.

### Implications and limitations of the study

Two-step cluster analysis examines a set of data by considering important interactions among attributes adherent to the set of data to reduce multidimensionality of data while discovering more relevant homogenous groups within heterogeneous set of data of HM(s). NF episodes among acute leukemias, in particular AML (*n* = 215, 57.8%), where constituting the majority of the episodes and sharing the same type of HM, we believed that they might have the impact on the results of the present study using two-step cluster analysis. NF episodes among AML in clusters 2, 3 and 4 resulted from cluster analysis in this study might also offer insights for future investigations or exploration of data on AML cases, such as, analyzing AML cases if any differences across groups or worthy of conducting cluster analysis on AML cases.

Regular institution surveillance appears in few, placing the responsibility on specialists to review the appropriate use of prophylaxis that has apparently been consolidated by expert panels while enquiring the impact of antimicrobial resistance. To combat risk for microbial-resistant strains because of inappropriate use of prophylaxis, establishing trial studies by active and repeated parameter assessments to signify possible low-risk HM population during the post-chemotherapy stage, even prior to the onset of fever, and thereby identifying needless administration of antimicrobial prophylaxis might be considered as an alternative evidence-based strategy. These active and repeated assessments might include frequent monitoring of patients’ vital sign parameters, and active checking of laboratory bio-indicators such as inflammatory markers and microbiological isolates from cultures. Active assessments might serve to mount a reliable diagnostic of pathogen and sepsis to secure an appropriate choice for the administration of antimicrobial prophylaxis. However, to date, regular comprehensive laboratory monitoring of bio-indicators prior to the onset of fever is less available in institutions, making real-time laboratory assessment a challenge. Our present study has limitations. It was retrospective in nature, since it was less likely that a considerable sample size could be recruited from the HM post-chemotherapy NF population. There were fewer characteristics or outcome parameters (e.g., deaths and microbiological isolates), which limited the ability to obtain significant results for analyses, including group comparisons as well as retrospective and prospective data comparisons. As this study was part of a larger observatory study with objectives, we did not collect data in details for the resistance of the pathogens, which limited the knowledge on dynamics/evolution of the resistance during the study period. Galactomannan assay and Beta-D-Glucan test have drawbacks of making false positive diagnosis of fungal infections, although these tests are noninvasive methods and have the advantages to aid in the assessment of patients with fungal infections [25, 26]. We considered that we would not neglect the positive results of these tests as fungal infections being the major infectious cause were found in a significant incidence among patients with HM. Yet, we acknowledged that there would have the possibility of over estimating cases with fungal infections in our study due to the diagnostic challenges of the tests, and that the study findings should be interpreted with caution.

## Conclusions

Our study showed that the occurrences of MDBIs and MDFIs were almost equal, with gram-negative bacteria being slightly predominant. Cluster analysis yielded four distinct clinical phenotype cluster groups, which might be meaningful to signify different risk patterns. There might have cases that were possibly in situations of low-risk cases and/or clinically diagnosed infections where microbiological isolates could not be identified in the laboratory cultures. Periodic institutional surveillance, coupled with establishing trial studies by active clinical parameter assessments, is essential in the management of NF in HM.

## Data Availability

Data was collected based on available medical records. All of the data was analyzed anonymously.

## Abbreviations

NF: Neutropenic fever
HM: Hematological malignancy
MDIs: Microbiological diagnosed infections
MDBIs: Microbiological diagnosed bacterial infections
MDFIs: Microbiological diagnosed fungal infections
CRP: C-reactive protein
PCT: Procalcitonin
Med: Median
IQR: Inter-quartile range
AIC: Akaike’s information criterion
E.coli: Escherichia coli
MRSA: Methicillin-resistant staphylococcus aureus
n: Sample size
AML: Acute myeloid leukemia
CLL: Chronic lymphocytic leukemia
ANC: Absolute neutrophil count
MDS: Myelodysplastic syndromes
MPD: Myeloproliferative disorders
ALL: Acute lymphocytic leukemia
CML: Chronic myeloid leukemia
NHL: Non-Hodgkin lymphoma
HL: Hodgkin lymphoma
%: Percentage of the total
*f*: Frequency counts
K-W test: Kruskal–Wallis test
MWU: Mann–Whitney U test
